# The Adjuvant Effect of Ambient Particulate Matter Is Closely Reflected by the Particulate Oxidant Potential

**DOI:** 10.1289/ehp.0800319

**Published:** 2009-03-11

**Authors:** Ning Li, Meiying Wang, Lori A. Bramble, Debra A. Schmitz, James J. Schauer, Constantinos Sioutas, Jack R. Harkema, Andre E. Nel

**Affiliations:** 1 Division of NanoMedicine, Department of Medicine; 2 Southern California Particle Center, University of California at Los Angeles, Los Angeles, California, USA; 3 Department of Pathobiology and Diagnostic Investigation, College of Veterinary Medicine, Michigan State University, East Lansing, Michigan, USA; 4 Department of Civil and Environmental Engineering, University of Wisconsin, Madison, Wisconsin, USA; 5 Department of Civil and Environmental Engineering, University of Southern California, Los Angeles, California, USA

**Keywords:** adjuvant, allergic inflammation, allergic sensitization, ambient ultrafine particles, asthma, oxidant potential, oxidative stress, redox-active organic chemicals, T_H_2 immune response

## Abstract

**Background:**

It has been demonstrated that ambient particulate matter (PM) can act as an adjuvant for allergic sensitization. Redox-active organic chemicals on the particle surface play an important role in PM adverse health effects and may determine the adjuvant effect of different particle types according to their potential to perturb redox equilibrium in the immune system.

**Objectives:**

We determined whether the adjuvant effect of ambient fine particles versus ultrafine particles (UFPs) is correlated to their prooxidant potential.

**Methods:**

We have established an intranasal sensitization model that uses ambient PM as a potential adjuvant for sensitization to ovalbumin (OVA), which enhances the capacity for secondary OVA challenge to induce allergic airway inflammation.

**Results:**

UFPs with a greater polycyclic aromatic hydrocarbon (PAH) content and higher oxidant potential enhanced OVA sensitization more readily than did fine particles. This manifests as enhanced allergic inflammation upon secondary OVA challenge, leading to eosinophilic inflammation and mucoid hyperplasia starting at the nasal turbinates all the way down to the small pulmonary airways. The thiol antioxidant *N*-acetyl cysteine was able to suppress some of these sensitization events.

**Conclusions:**

The adjuvant effects of ambient UFP is determined by their oxidant potential, which likely plays a role in changing the redox equilibrium in the mucosal immune system.

Ambient particulate matter (PM) exposure as a result of fossil combustion activity and vehicular traffic is associated with increased cardio-respiratory morbidity and mortality (Delfino et al. 2005; Nel et al. 1998; Riedl 2008; Sun et al. 2005). This includes increased morbidity as a result of allergic disorders such as asthma and allergic rhinitis (Bernstein et al. 2004; D’Amato et al. 2005; Lippmann 2007). This is evidenced by epidemiologic studies demonstrating an association between the incidence of allergic diseases and the residential freeway proximity as well as an increase in asthma flares after a sudden surge of ambient PM levels (Bernstein et al. 2004; Samet et al. 2000). Although the acute asthma flares could relate to an exacerbation of existing airway inflammation or airway hyperreactivity, PM could also exert an adjuvant effect in the respiratory tract that could lead to an increased prevalence of allergic disease (de Haar et al. 2006; Diaz-Sanchez et al. 1997, 1999; Inoue et al. 2005; Kleinman et al. 2007; Matsumoto et al. 2006; Nel et al. 1998).

PM adjuvant effects have been demonstrated in both animal and human studies (Diaz-Sanchez et al. 1997; Gilliland et al. 2004; Kleinman et al. 2007; Matsumoto et al. 2006; Steerenberg et al. 2003a, 2003b; Stevens et al. 2008; Whitekus et al. 2002). Although in humans it has been shown that intranasal instillation of diesel exhaust particles (DEP) could enhance ragweed-induced immunoglobulin E (IgE) and interleukin-4 (IL-4) production, results from animal studies have demonstrated that low-dose challenge by aerosolized inhalation or intratracheal instillation could enhance allergic sensitization to an experimental allergen such as ovalbumin (OVA) (Diaz-Sanchez et al. 1997; Gilliland et al. 2004; Matsumoto et al. 2006; Steerenberg et al. 2003a, 2003b; Stevens et al. 2008; Whitekus et al. 2002). Similar findings in studies using ambient PM exposure, including the recent Los Angeles study, have demonstrated that the inhalation of concentrated ambient PM near a busy freeway could increase antigen-induced airway responses in mice (Kleinman et al. 2007).

Two key issues regarding the adjuvant effect of PM are the mechanism of the adjuvant effect and the PM components that are responsible for this effect. Although a variety of mechanisms have been shown to explain the adverse respiratory effects of PM, one possibility that has emerged is that the organic chemical fraction of PM could play an important role in the adjuvant effect through the ability to generate reactive oxygen species (ROS) in the respiratory tract (Li et al. 2003a, 2008; Nel et al. 2006). Organic DEP extracts are capable of changing the redox equilibrium of dendritic cells (DCs) in the mucosal immune system such that their ability to present OVA to T-cells results in a polarized immune response in which there is a decrease in T helper 1 (T_H_1) and increase in T helper 2 (T_H_2) immunity (Chan et al. 2006). This leads to the prediction that the prooxidant potential of PM plays a role in determining adjuvant effect. This hypothesis has not yet been formally tested in an *in vivo* model for PM adjuvant effects. In fact, most of the animal studies to date have used poorly calculated PM doses that far exceed the real-life exposure amounts and do not address the mechanism of the adjuvant effect (Ichinose et al. 2004; Inoue et al. 2005; Steerenberg et al. 2003a, 2003b). Thus, we aimed to determine whether there is a positive correlation between the adjuvant effect of ambient concentrated PM and their content of redox cycling organic chemicals.

In this study, we used a murine intranasal sensitization model and a precise amount of size-fractionated ambient PM collected by particle concentrators in the Southern California Particle Center to determine how this concentrated PM may contribute to an adjuvant effect through intranasal administration in a murine OVA sensitization model (Li et al. 2003a). This model allowed us to compare ultrafine particles (UFPs) with an aerodynamic diameter < 0.15 μm with a mixed atmosphere of fine, < 2.5 μm particles, which in this report we refer to as fines and ultrafines (F/UF). The end points that we used to evaluate the adjuvant effect of ambient PM included nasal and pulmonary inflammation as the measurement of OVA-specific IgG_1_ and IgE in the blood. We also used morphometric analysis of mucosubstances and eosinophils to show that the allergic sensitization leads to an allergic inflammatory response in both upper and lower airways. Finally, we measured IL-5 and IL-13 production as signature cytokines for T_H_2 allergic inflammatory responses. We found that the enhanced *in vivo* adjuvant effects of the concentrated ambient UFP correlate with a higher *in vitro* oxidant potential and higher content of redox-cycling organic chemicals.

## Materials and Methods

### Reagents

See Supplemental Material (available online at http://www.ehponline.org/docs/2009/0800319/suppl.pdf) for information.

### Ambient PM collection and endotoxin detection

We used the Versatile Aerosol Concentrator Enrichment System (VACES) to collect ambient atmospheres composed of PM < 2.5 μm (fine/ultrafine; F/UF) as well as PM < 0.15 μm (ultrafine particles; UFPs) in downtown Los Angeles (Li et al. 2002a, 2003b; Sioutas et al. 2005). The collection site was about 200 m from a major freeway, where most traffic consists of passenger cars and diesel trucks. The specific details about the characteristics and composition of the PM collected near Interstate highway 110 has been previously reported (Sioutas et al. 2005). The particles were collected in sterile deionized water from 10:00 hr to 17:00 hr Monday through Friday using an impinger (SKC West Inc., Fullerton, CA; Li et al. 2003b). The samples designated F/UF#1 and UF#1 were collected side by side in January 2007, and the collection of UF#2 took place at the same site in September 2006. Although the F/UF atmosphere includes some UFPs, on a per mass basis the UFP atmosphere includes a much higher content of concentrated < 0.15 μm PM. Moreover, the UFP collections included PM with a much larger surface area and higher fractional organic carbon (OC) content than did the F/UF atmosphere (Araujo et al. 2008). All concentrated ambient particles (CAPs) contained low levels of endotoxin [see Supplemental Material, Table 1 (http://www.ehponline.org/docs/2009/0800319/suppl.pdf)].

### Allergic sensitization and PM exposure

We obtained 6- to 8-week-old female BALB/c mice from Charles River Laboratories (Hollister, CA). Mice were housed under standard laboratory conditions approved by the University of California at Los Angeles (UCLA) Animal Research Committee. We used endotoxin-free OVA as the allergen for allergic sensitization. On day 1, mice in the PM exposure group received intranasal instillation of 0.5 μg of the PM suspension in a total volume of 50 μl. Mice in the OVA-only and control groups received the same volume of saline alone. On day 2, animals in the PM exposure groups received intranasal instillation of 0.5 μg PM together with 10 μg OVA, whereas those in the OVA and control groups received OVA and saline only. Intranasal instillations were repeated on days 4, 7, and 9. In a different experiment, we administered the thiol antioxidant *N*-acetyl cysteine (NAC) at a dose of 320 mg/kg through intraperitoneal injection 4 hr before each of the intranasal instillations on days 1, 2, 4, 7, and 9. We have previously demonstrated the anti-oxidant properties of this agent in animal and *in vitro* studies (Whitekus et al. 2002). After animals were rested, we then challenged them with 1% OVA aerosol for 30 min in a nebulizer on days 21 and 22 (Hao et al. 2003), and sacrificed them on day 23. All animal procedures were approved by the UCLA Animal Research Committee. All mice were treated humanely, with regard for pain and suffering, by strictly following the guidelines set by UCLA and National Institutes of Health.

### Animal necropsy, sample collection, and analysis

Mice were anesthetized by intra-peritoneal injection of pentobarbital. We performed blood and bronchoalveolar lavage (BAL) collections and differential BAL cell counts as previously described (Hao et al. 2003). The right lung was collected and stored in liquid nitrogen for future analyses. The left lung was expanded with 10% buffered formalin before processing it for histologic staining and microscopy. We measured plasma OVA-specific IgG_1_ (OVA-IgG_1_) and IgE (OVA-IgE) by enzyme-linked immunosorbent assay (ELISA) (Hao et al. 2003). Quantification of nine proinflammatory cytokines [tumor necrosis factor-α (TNF-α), interferon-γ (IFN-γ), IL-4, IL-5, IL-6, IL-13, keratinocytes chemoattractant (KC), monocyte chemotactic protein-1 (MCP-1), and macrophage inflammatory protein 1 (MIP-1α)] in the BAL fluid was determined with the Cytometric Bead Array Mouse Inflammation Kit according to the manufacturer’s instructions (BD BioSciences, San Diego, CA).

### Nasal and lung tissue preparation for morphometry and immunohistochemistry

Tissues were removed from the nasal and intrapulmonary axial airway sections as shown in Supplemental Material, Figure 1 (available online http://www.ehponline.org/docs/2009/0800319/suppl.pdf). Nasal and lung tissues were prepared for morphometry and immunocytochemistry as described in detail in the Supplemental Material (available online at http://www.ehponline.org/docs/2009/0800319/suppl.pdf).

### Morphometric analysis of mucosubstances and eosinophils in nasal and pulmonary airways

Quantitative analyses of stored muco-substances and eosinophils in the surface epithelium lining of the maxilloturbinates in the proximal nasal section T1 and of the proximal and distal axial airways in the lung (airway generations 5 and 11, respectively) were estimated using computerized image analysis and standard morphometric techniques, as previously reported [see Supplemental Material, Figure 1 (http://www.ehponline.org/docs/2009/0800319/suppl.pdf)] (Farraj et al. 2003; Harkema et al. 1997). Supplemental Material (available online at http://www.ehponline.org/docs/2009/0800319/suppl.pdf) describes the methods in detail.

### Induction of intracellular oxidative stress

We used heme oxygenase-1 (HO-1) protein expression in the murine macrophage cell line (RAW 264.7) as a biological oxidative stress marker that reflects the prooxidant potential of concentrated ambient PM (Li et al. 2000, 2002a, 2003b). We performed Western blotting for HO-1 expression as previously described (Li et al. 2002a, 2002b, 2003b).

### Dithiothreitol assay

We determined the abiotic assessment of the oxidant potential of CAPs by the dithiothreitol (DTT) assay. This assay quantitatively measures superoxide production by redox cycling organic chemicals such as quinones (Cho et al. 2005; Li et al. 2003b). We have also previously shown that introducing fractionated organic DEP extracts into this assay demonstrates that most of the redox cycling activity resides in the polycyclic aromatic hydrocarbon (PAH)–enriched and quinine-enriched silica gel fractions (Li et al. 2000).

### PM composition and chemical analysis

We used quartz and Teflon filters for CAP collection in parallel with the impinger samples. These filters were used to analyze PM chemical composition and PAH content as described in the Supplemental Material (available online at http://www.ehponline.org/docs/2009/0800319/suppl.pdf) (Li et al. 2003b).

### Statistical analysis

We express results as mean ± SE. Differences among groups were evaluated by analysis of variance and the Student *t*-test was used to distinguish between pairs of groups. We considered *p* < 0.05 statistically significant. Pearson correlation coefficients were calculated to examine associations between the oxidant potential and the chemical content of PM (Li et al. 2003b).

## Results

### Establishment of an allergic sensitization model to demonstrate the adjuvant effect of ambient UFP

**Figure 1:**
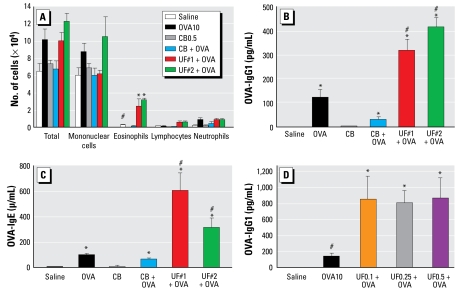
The adjuvant effect of ambient UFP on allergic sensitization. (*A*) Ambient UFP (UF#1 and UF#2, 0.5 μg/instillation) increased OVA-induced allergic inflammation in the lung. (*B*) Enhanced OVA-IgG_1_ production by 0.5 μg UFP instillation. (*C*) Enhanced OVA-IgE production by 0.5 μg UFP instillation. (*D*) Administration of 0.1 μg of UFP enhanced OVA-IgG_1_ production in parallel with other effects on allergic sensitization. *p < 0.05 compared with control; #*p* < 0.05 compared with OVA alone.

Although most of the published *in vivo* studies that have addressed the adjuvant effects of PM have used DEP, few have looked at ambient PM. We therefore set out to develop an animal model to test the adjuvant effect of ambient PM collected by particle concentrators in downtown Los Angeles. We collected two independent sets of ambient UFPs (UF#1 and UF#2) near Interstate highway 110 and used them in an OVA intranasal instillation model. In the initial setup, the mice received saline, OVA (10 μg), or OVA (10 μg) plus UFP (0.5 μg) for allergic sensitization. To exclude the possibility that the nanosized carbon core of the UFP was promoting the adjuvant effect, we also used an equivalent amount of ultrafine carbon black particles (CB) as a control. BAL analysis showed that both UF#1 and UF#2 were quite effective in enhancing OVA sensitization. Compared to saline, OVA alone, CB alone, or CB plus OVA, UFP plus OVA induced a statistically significant increase in the BAL eosinophil count (*p* < 0.05; Figure 1A). Extensive testing of UFPs alone did not reveal an effect on eosinophilic inflammation. The enhanced airway inflammation was accompanied by significantly increased OVA-specific IgG_1_ (OVA-IgG_1_) and IgE (OVA-IgE) in the plasma (Figure 1B, C). These Ig classes reflect T_H_2 immunity. Both UFP collections yielded similar results. CB alone or in combination with OVA failed to exert an effect. Additional dose–response studies using UF#1 showed that as little as 0.1 μg UFP could elicit an adjuvant effect as determined by the OVA-IgG_1_ response (Figure 1D).

**Figure 2:**
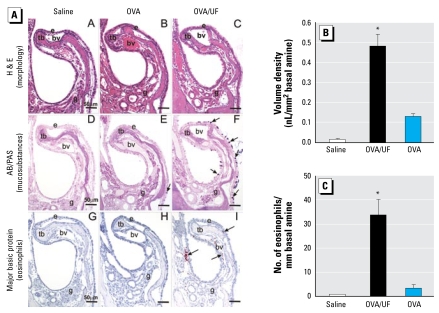
Histopathology and morphometry of nasal maxilloturbinates. Abbreviations: AB/PAS, Alcian blue-Periodic acid Schiff double stain; bv, blood vessel in subepithelial lamina propria; g, nasal lateral glands in lamina propria; H&E, hematoxylin and eosin stain; tb, turbinate bone. (*A*) Morphologic features of allergic rhinitis in OVA/UFP-exposed animals. Bars = 50 μm. Arrows in (*E* and *F*) depict AB/PAS-stained mucosubstances in airway eoithelium. Arrows in (*I*) depict eosinophils containing major basic protein. (*B* ) Quantification of mucosubstances in the surface epithelium shown as volume density of intraepithelial mucosubstances (mean ± SE). (*C*) Numeric eosinophil densities in T1 nasal section. Bars represent group means (n = 4–6 mice) ± SE. *p **=** 0.05 compared with saline or OVA alone.

We determined the extent of the allergic sensitization by nasal and pulmonary histopathology and airway morphometry. Only mice exposed to the UFP/OVA combination exhibited allergic inflammation in the nasal mucosa (Figure 2A). These changes were restricted to intranasal regions lined by transitional or respiratory epithelium (Figure 2A). No changes occurred in the olfactory epithelium (data not shown). For a full account of the nasal sites that we analyzed, see Supplemental Material, Figure 2 (available online at http://www.ehponline.org/docs/2009/0800319/suppl.pdf). The principal pathologic changes were mucous cell metaplasia/hyperplasia of airway epithelium accompanied by a mixed inflammatory cell infiltration in the underlying lamina propria (Figure 2A) The infiltrates were composed of eosinophils, mononuclear cells (lymphocytes and plasma cells), and a lesser number of neutrophils. Figure 2A illustrates exposure-related mucous cell metaplasia and eosinophil influx in the mucosa overlying the maxillo-turbinates. In UFP-exposed mice, there was a markedly greater amount of mucosubstances in the nasal transitional epithelium lining the maxilloturbinates compared with those in the control or OVA-alone groups [Figure 2B; see Supplemental Material, Figures 1, 3 (http://www.ehponline.org/docs/2009/0800319/suppl.pdf)]. Morphometric determination of numeric cell density showed a significant increase of eosinophils at the nasal mucosa biopsy sites [Figure 2C; see Supplemental Material, Figures 1, 3 (http://www.ehponline.org/docs/2009/0800319/suppl.pdf)].

**Figure 3:**
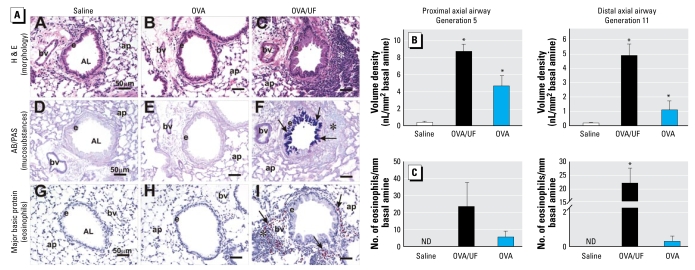
Histopathology and morphometry of the left lung lobe. Abbreviations: AB/PAS, Alcian blue-Periodic acid Schiff double stain; AL, airway lumen; ap, alveolar parenchyma; bv, blood vessel; e, airway surface epithelium. (*A*) Morphologic features of allergic lung inflammation in OVA/UFP-treated mice. Tissues from preterminal bronchioles were analyzed for mucosubstances in mucous cells and major basic protein in eosinophils. Bars = 50 μm. Asterisks depict peribronzcholar mixed inflammatory cell infiltrate composed of lymphocytes, plasma cells and eosinophils. Arrows in (*F*) depict AB/PAS-stained mucosubstances in airway epithelium. Arrows in (*I*) depict eosinophils conatining major basic protein. (*B*) Quantification of mucosubstances in the surface epithelium lining the proximal and distal axial airways in the lung shown as volume density of intraepithelial mucosubstances (mean ± SE; *n* = 6/group). (*C*) Numeric densities of intramural eosinophils in the proximal and distal axial airways shown as group means ± SE (*n* = 4–6 mice). *p **=** 0.05 compared with saline or OVA alone.

The nasal mucosa and BAL changes were accompanied by histologic evidence of eosinophil and mononuclear cell infiltration around small airways in OVA/UFP-sensitized mice (Figure 3A). Similar to the changes in the nose, the major morphologic changes in the lungs of OVA/UF#1-treated mice consisted of marked mucous cell metaplasia in the surface epithelium lining the conducting airways (large- and small-diameter bronchioles) plus an associated mixed inflammatory cell influx consisting mainly of eosinophils, lymphocytes, and plasma cells in the interstitial tissues surrounding these airways (Figure 3A). Airway lesions were most severe in the main axial airways, but were also present to a slightly lesser degree in the small-diameter, terminal bronchioles of the mice exposed to both OVA and UFP. Along the axial airways, the volume densities of intraepithelial muco-substances in the proximal and distal generations (5 and 11) were approximately 22 and 24 times greater, respectively, than those measured at the same airway generations in saline-instilled control mice (Figure 3B).

Mice exposed to OVA only exhibited definitive but milder epithelial and inflammatory alterations in the large-diameter, preterminal and small-diameter, terminal bronchioles (Figure 3). Moreover, the volume densities of mucosubstances in the proximal axial airways (generation 5) of OVA/UF#1-treated mice were approximately twice that of OVA-treated mice (Figure 3B). In the distal axial airway (generation 11), OVA/UF#1-treated mice had almost five times more intraepithelial mucosubstances compared with those in OVA-alone mice (Figure 3B). Consistent with allergic inflammation, morphometric analysis of numeric cell densities demonstrated a significant increase of intramural eosinophils in both proximal and distal axial airways (Figure 3C).

**Figure 4:**
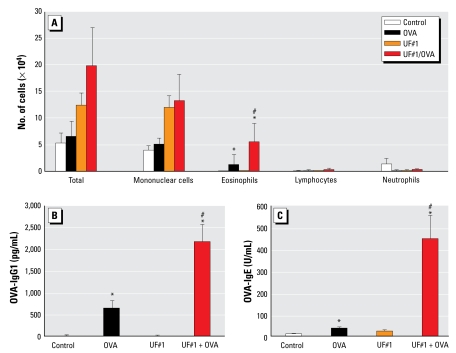
UFP alone failed to elicit any proinflammatory effect. In the absence of OVA, intranasal instillation of UF#1 did not have any effect, whereas a combination of UF#1 and OVA induced significant increase in eosinophil infiltration (*A*) and OVA-specific IgG_1_ (*B*) and IgE production (*C*). *p < 0.05 compared with control. #*p* < 0.05 compared with OVA.

UFP alone did not exert any effect in the lung. Figure 4 shows that, although 0.5 μg UF#1 alone had no impact, the same particle batch did exert an adjuvant effect when combined with OVA. This resulted in eosinophilic inflammation and increased OVA-specific IgG_1_ and IgE antibody production. We obtained similar results with UF#2 (Figure 1).

### The adjuvant effect of UFP is related to their content of prooxidative organic chemicals

**Figure 5:**
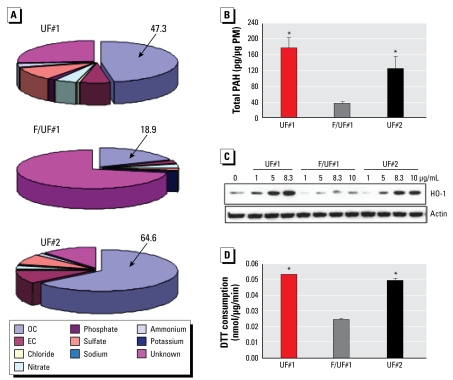
Correlation between the organic chemical content of UFP and its oxidant potential. (*A*) Chemical analysis of UFP and < 0.25 μm collection. EC, elemental carbon. (*B*) Total content of 17 signature PAHs in UFP and F/UF collection. (*C*) Biotic assay showing HO-1 expression in RAW 264.7 cells as determined by immunoblotting. (*D*) Abiotic measurement by DTT assay to compare the redox potential of UFP and < 0.25 μm collection. *p < 0.05 compared with F/UF#1.

We have previously shown that combustion particles such as DEP have a high content of redox cycling organic chemicals in the PAH-enriched aromatic and the quinone-enriched polar fractions (Li et al. 2000). Because UFPs mostly derive from combustion sources, these particles (UF#1 and UF#2) also exhibit high OC contents (47.3% and 64.6% on a per mass basis, respectively) compared with the mixed particle atmosphere (F/UF#1, 18.9%; Figure 5A). Moreover, measurement of the PAH content, which serves as a proxy for the presence of the redox cycling OC chemicals, showed that on a per mass basis the content of signature PAHs in the UFP is considerably higher than in the F/UF#1 collection [Figure 5B; see Supplemental Material, Figure 3 (http://www.ehponline.org/docs/2009/0800319/suppl.pdf)] (Araujo et al. 2008). The PAH profile is typical of combustion particles in which the partitioning of lower molecular weight PAHs is typical of a winter collection [see Supplemental Material, Figure 3 (http://www.ehponline.org/docs/2009/0800319/suppl.pdf)] (Li et al. 2002a). For instance, both UFP collections contained significantly larger amounts of PAHs such as benzo[*a*]pyrene (BaP) that can be metabolically converted to redox cycling quinones such as the benzo[*a*]pyrene quinones (BaP-Q). To determine whether the observed adjuvant effect can be correlated to differences in the oxidative stress potential of the UFP and < 2.5 μm collections, we performed abiotic and biotic assays that reflect their oxidant potential (Li et al. 2003b). HO-1 expression is a sensitive biotic assay for PM-induced oxidative stress (Li et al. 2000, 2002a, 2002b, 2003a, 2003b). Immunoblotting revealed that both UF#1 and UF#2 induced more robust HO-1 expression in the macrophage cell line RAW 264.7 compared with the F/UF#1 (Figure 5C). We based the abiotic assay on the oxidation of DTT by redox cycling organic chemical compounds such as quinones (Cho et al. 2005; Li et al. 2003b). This assay demonstrated that the DTT consumption of UF#1 and #2 was > 2-fold higher than F/UF#1 (Figure 5D, *p* < 0.05). Calculation of the Pearson correlation coefficient confirmed that the higher PAH content of UFP correlates with HO-1 and DTT results (Figure 5B–D, Table 1). Although similar analyses could not be carried out in live animals in the early stage of the experiment, we have previously demonstrated that DEP induce oxidative stress in mouse lungs as determined by a carbonyl protein assay (Whitekus et al. 2002).

**Figure 6:**
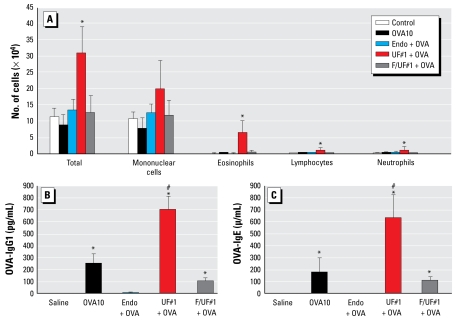
The adjuvant effect is a unique feature of UFP. Ambient fine PM and UFP were simultaneously collected and tested for their adjuvant effects. Although UFP reproduced previous results, F/UF had no effect. (*A*) BAL analysis showing the enhancing effect of UFP on eosinphilic inflammation in the lung. (*B*) Increased OVA-IgG1 production by UFP. (*C*) Increased OVA-IgE production by UFP. *p **<**0.01 compared with control; #*p* < 0.01 compared with OVA alone or F/UF#1+OVA.

UF#1 differed significantly from F/UF#1 in its adjuvant effects in our intransal sensitization model (Figure 6). Although the < 2.5 μm PM (F/UF#1) failed to significantly boost eosinophilic inflammation or OVA-IgE and IgG_1_ responses, the UFP-only collection (UF#1) was associated with significant adjuvant effects (Figure 6A–C). Similar adjuvant effects could not be achieved by combining OVA with endotoxin at levels similar to those present in ambient PM (Figure 6). For a discussion of endoxin levels of PM and OVA see Supplemental Material (available online at http://www.ehponline.org/docs/2009/0800319/suppl.pdf).

Analysis of proinflammatory cytokines and chemokines in the BAL fluid provided further evidence of UFP adjuvant effects *in vivo* (Table 2). Although F/UF#1 had little effect, UF#1 significantly enhanced the induction of T_H_2 cytokines (IL-5 and IL-13) as well as several other proinflammatory mediators (TNF-α, IL-6, KC, MCP-1, and MIP-1α) on OVA challenge (Table 2). Endotoxin had no impact. Interestingly, the T_H_1 cytokine IFN-γ did not change in any of the groups (Table 2).

### Use of a thiol antioxidant to suppress the adjuvant effect of UFP

**Figure 7:**
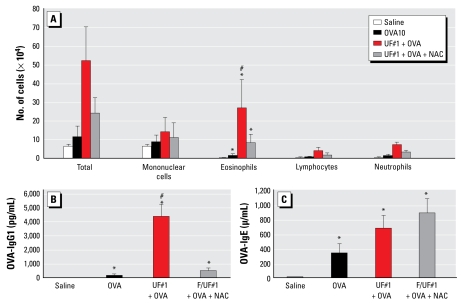
Partial inhibition of the UFP adjuvant effect by the thiol antioxidant NAC. (*A*) Pretreatment with NAC suppressed UFP-enhanced eosinophilia in the lung. (*B*) Inhibition of the UFP effect as determined by OVA-IgG_1_ production. (*C*) NAC failed to inhibit UFP-stimulated OVA-IgE production. #*p* < 0.05 compared with control. *p < 0.05 compared with OVA alone or UF#1+OVA+NAC.

We have previously demonstrated that thiol antioxidant NAC is effective in suppressing the adjuvant effect of DEP *in vivo* (Whitekus et al. 2002). NAC accomplishes this effect by serving as a glutathione precursor and oxygen radical scavenger and through direct covalent coupling to redox cycling organic chemicals such as quinones (Xiao et al. 2003). Intraperitoneal administration of this agent 4 hr before the intranasal administration of UFP (days 1, 2 4, 7, and 9) significantly suppressed BAL eosinophils and OVA-specific IgG_1_ production (Figure 7A, B) but did not interfere with the OVA-IgE response (Figure 7C). The reason for this lack of the IgE response is unknown. We repeated the experiment, with the same result.

## Discussion

There is growing recognition that the redox chemistry of organic chemical compounds plays a crucial role in the biological effect of ambient PM (Araujo et al. 2008; Dellinger et al. 2001; Li et al. 2003a, 2003b, 2008; Nel et al. 2001; Squadrito et al. 2001). Moreover, increased polar resolution of silica gel columns that were prior loaded with an organic DEP extract has shown that most of the redox cycling activity in the OC fraction segregates with the PAH-enriched aromatic as well as the quinone-enriched polar fractions (Li et al. 2000). Although a number of animal models have been established to study the adjuvant effect of DEP on allergic sensitization, there are few data for ambient PM (Ichinose et al. 2004; Kleinman et al. 2007; Matsumoto et al. 2006; Samuelsen et al. 2008; Song et al. 2008). In addition, previously published animals models for evaluating PM adjuvant effects have limitations in sensitivity, reproducibility, and *in vivo* dosimetry. Here we show that ambient UFP collected by a particle concentrator act as an adjuvant for allergic sensitization. Moreover, the adjuvant effects were closely correlated to the higher oxidant potential of UFP and could be partially blocked by NAC administration. We demonstrate that the allergic sensitization by UFP leads to extensive excitation of allergic inflammation in the upper as well as lower respiratory tract upon secondary OVA challenge. This presents another unique feature of our study. This allergy model could be useful for comparing ambient PM that varies in oxidant potential as a result of differences in the source, collection site, season, and ambient temperature. In this regard, we have previously demonstrated that the oxidant potential of UFP varies with season and temperature, likely as a result of influencing the partitioning of redox cycling PAHs onto the particle surface (Li et al. 2002a).

We developed a murine model in which nanogram quantities of ambient UFP can be used to achieve allergic sensitization. Intranasal instillation with as little as 100–500 ng UFP is sufficient to enhance allergic sensitization to OVA (Figure 1D). This allergic sensitization manifested as significantly increased eosinophilic airway inflammation in parallel with increased OVA-IgG_1_ and OVA-IgE production (Figure 1A–C). We could not elicit similar responses with the same dose of < 2.5 μm PM (F/UF#1; Figure 6). Although the latter collection includes some UFPs, the fractional composition is very different from the UFP collection (Figure 5) (Li et al. 2002a). Although as much as 65% of the UFP weight derives from OC compounds, the OC content of the mixed atmosphere is much smaller (Figure 5). These differences reflect the higher content of UFPs in the < 0.15 μm atmosphere. From a toxicologic perspective, this could mean the redox active organic compounds on the smaller particles could be more bioavailable because of their large surface area. We propose that PAHs are a good proxy for the specific redox cycling chemicals in the OC fraction that is responsible for the biological effect (Li et al. 2000, 2003a, 2003b; Nel et al. 1998; Stevens et al. 2008). This also agrees with the excellent correlation between the PAH content of UFP and their ability to induce DTT consumption and HO-1 expression (Figure 5, Table 1). PAHs such as BaP can be metabolically converted to BaP-Q, a series of potent redox cycling quinones that are responsible for oxidative stress (Li et al. 2000; Mass and Kaufman 1979). In fact, BaP has been shown to act as an adjuvant for allergic sensitization in animal studies (Kadkhoda et al. 2004, 2005). CB, which lacks redox cycling compounds and consists mostly of elemental carbons, did not exert an adjuvant effect (Figure 1).

Organic chemical compounds such as oxy-PAHs and quinones are relevant organic chemical species in terms of PM redox chemistry and ROS generation (Bonvallot et al. 2001; Cho et al. 2005; Kumagai et al. 1997; Monks et al. 1992; Penning et al. 1999). A recent study has demonstrated that PAH coated onto PM_2.5_ could induce gene expression of cyto-chrome P450 (CYP) 1A1, CYP2E1, NADPH quinone oxydo-reductase-1, and glutathione *S*-transferasepi 1 and mu 3 in human alveolar macrophages, suggesting the formation of biologically reactive metabolites and the role of carbonaceous core of PM as a physical carrier (Saint-Georges et al. 2008). Quinones act as catalysts that produce ROS and may be key compounds in PM toxicity along with transition metals. Quinones can be formed as by-products of diesel fuel combustion as well as from metabolic conversion of PAH in the lung (Bonvallot et al. 2001; Cho et al. 2005; Kumagai et al. 1997; Monks et al. 1992; Penning et al. 1999). Although the < 2.5 μm collection (F/UF#1) contained UFPs and redox-active chemicals, the lower PAH content and ability to promote oxidative stress effects could explain its lack of an adjuvant effect (Li et al. 2003a, 2003b; Xiao et al. 2003). These particles cannot induce the equivalent of a tier 1 oxidant response in our hierarchical oxidative stress model (Wang et al. 2005; Xia et al. 2004; Xiao et al. 2003). That level of oxidative stress, however, is mostly a protective response that is mediated by phase 2 enzyme expression and does not amount to the induction of proinflammatory effects (tier 2) that we postulate is required to modify the effect of antigen presentation *in vivo* so as to skew the immune response to T_H_2 cytokine production (Chan et al. 2006). In contrast, UFPs do have the ability to achieve this adjuvant threshold. We propose that a similar threshold is required for existing *in vivo* PM effects. Although the tenets of the hierarchical oxidative stress model still needs to be confirmed *in vivo*, the biological significance of oxidative stress in PM adjuvant effects was previously confirmed by the use of NAC (Li et al. 2002b; Whitekus et al. 2002). We further confirmed this in the present study by demonstrating that NAC was able to suppress eosinophilic inflammation and OVA-IgG_1_ production (Figure 6).

The physical and chemical properties of UFP play important roles in particle deposition in the respiratory system and translocation to the extrapulmonary tissues (BeruBe et al. 2007; Kreyling et al. 2006; Oberdorster 2001; Peters et al. 2006). The small size and large surface area of these particles may contribute in an important way to their adjuvant effects. Their small size allows UFP to penetrate deeply into the lung and to enter epithelial and antigen-presenting cells, which could be responsible for promoting the adjuvant effect (Chan et al. 2006; Li et al. 2002b, 2003a, 2003b, 2008). The large surface area of UFP allows these particles to carry twice the cargo of organic chemical compounds, which we show are responsible for inducing oxidative stress responses. Thus, UFP likely carry a higher content of bioavailable redox-active compounds on a per mass basis than do the larger particles (Araujo et al. 2008).

Little is known about the immunologic basis for PM adjuvant effects. Prooxidative PM can skew the immune response toward T_H_2 differentiation through an impact on DC signaling pathways (Porter et al. 2007). One explanation is that PM enhance DC antigen uptake and costimulatory receptor expression, leading to increased IL-13 and decreased IFN-γ production in T-cells (Porter et al. 2007). This is in keeping with the demonstration of an increased IL-5 and IL-13 content in BAL fluid of animals sensitized with OVA plus UFP (Table 2). Another possibility is that the generation of oxidative stress by organic PM chemicals induce Nrf2 expression, which interferes in IL-12 and IFN-γ production, thereby leading to decreased T_H_1 responses (Chan et al. 2006). Similar inhibitory effects on IFN-γ production have also been demonstrated in intact animals (Finkelman et al. 2004).

In summary, we have established a highly sensitive *in vivo* model system for studying the adjuvant effect of ambient PM on allergic sensitization. This model demonstrates that ambient UFPs, but not F/UF, can act as an adjuvant to promote T_H_2 polarization and to enhance allergic sensitization. The adjuvant effect of UFP is premised on redox chemistry, which is closely related to the prooxidative organic chemical content on these particles.

## Figures and Tables

**Table 1:** Analyses of Pearson correlation coefficient.

Comparison	Pearson correlation coefficient
OC vs. DTT	0.882
OC vs. HO-1^a^	0.943
Total PAH vs. DTT	0.967
Total PAH vs. HO-1^a^	0.997

a HO-1 band density was used to calculate the Pearson correlation coefficient.

**Table 2:** The effects of UFP on cytokine levels in the lung (mean ± SE).

Cytokine	Saline	OVA	Endo+OVA	UF#1+OVA	F/UF#1+OVA
TNF-α	1.63 ± 0.1	2.75 ± 0.2	3.08 ± 0.5	5.40 ± 0.5*	3.22 ± 0.6
IFN-γ	1.13 ± 0.0	1.13 ± 0.0	1.20 ± 0.1	1.48 ± 0.2	1.07 ± 0.2
IL-5	0.95 ± 0.6	1.98 ± 0.2	1.95 ± 0.7	13.5 ± 3.1*	2.78 ± 0.9
IL-4	0.00 ± 0.0	0.25 ± 0.3	0.00 ± 0.0	0.82 ± 0.4	0.00 ± 0.0
IL-13	1.25 ± 0.5	1.51 ± 0.6	1.77 ± 0.2	4.19 ± 0.8*	1.81 ± 0.2
KC	3.84 ± 0.3	8.17 ± 0.8	4.81 ± 0.8	12.8 ± 0.6*	5.51 ± 1.0
IL-6	0.56 ± 0.3	1.41 ± 0.1	1.34 ± 0.1	2.43 ± 0.3*	1.22 ± 0.3
MCP-1	0.00 ± 0.0	0.00 ± 0.0	0.00 ± 0.0	9.34 ± 3.2*	0.00 ± 0.0
MIP1-α	1.09 ± 0.4	1.99 ± 0.1	2.56 ± 0.4	4.25 ± 0.6*	2.18 ± 0.3

Endo, endotoxin. The amount of endotoxin was equal to that in the PM samples.

BAL fluid was obtained from the same mice as those in Figure 6. All cytokine concentrations were in picograms per milliliter .

* *p* < 0.05 compared with control and OVA.
